# Using Rapid Cycle Deliberate Practice to Up-Train Pediatric Providers for Adult COVID-19 Patients

**DOI:** 10.7759/cureus.18283

**Published:** 2021-09-25

**Authors:** Wendy L Van Ittersum, Stephanie A Estephan

**Affiliations:** 1 Pediatrics, Akron Children's Hospital, Akron, USA; 2 Pediatrics, Simulation, Akron Children’s Hospital, Akron, USA

**Keywords:** rapid cycle deliberate practice, simulation, interprofessional team training, covid-19, pre-briefing

## Abstract

The SARS CoV-2 pandemic brought unique challenges to healthcare workers and systems. Simulation teams improvised and scaled up to meet new educational needs. Children’s hospitals worked to address the many issues surrounding COVID-19, including how to prepare facilities and staff to care for adult patients in the event of COVID patient overflow. This technical report describes the use of the teaching method rapid cycle deliberate practice (RCDP) to train interprofessional teams unaccustomed to working together. We detail how sessions were developed and implemented, particularly noting the need for an extended pre-briefing to optimize psychological safety. The RCDP model allowed for a high level of interaction throughout the simulations and the incorporation of new knowledge “on the go” during the sessions.

## Introduction

As the uncertainty of COVID-19 swept through communities, our free-standing tertiary care academic children’s hospital prepared for the disaster. In the event that adult COVID patients overwhelm area hospital capacity, the hospital would need to care for these patients. The plan was for the intensive care unit (ICU) to expand coverage to the acute care floors. Given their residency training in adults, surgeons and anesthesiologists would staff these units supported by pediatric intensivists, with pediatric hospitalists providing a third tier of coverage. Acute care unit nurses would provide patient care supported by an ICU nurse. The hospital rapidly began to up-train pediatric personnel to care for adults, but immediately multiple challenges became evident. New teams, unacquainted with each other and their usual workflows, would be working together in foreign clinical environments. Additionally, transitioning acute care units into ICUs required bringing in new equipment, medications, and developing protocols, many of which underwent daily revision. Simulation offered an ideal platform to address these challenges.

## Technical report

Planning

As our simulation team began planning, the use of rapid cycle deliberate practice (RCDP) seemed an excellent fit. Rapid cycle deliberate practice involves frequently interrupting a scenario to give immediate feedback, troubleshooting if necessary, then “rewinding” and allowing learners the opportunity to correct the errors discussed [1,2]. Our training situation called for introducing and mastering new behaviors, revising existing choreography for specific situations, and sharing new performance guidelines, all of which fit well into an RCDP framework [2]. In this case, stopping the scenario would break learning into digestible pieces so participants felt less overwhelmed, allowing them to pause the scenario as questions arose.

**Figure 1:**
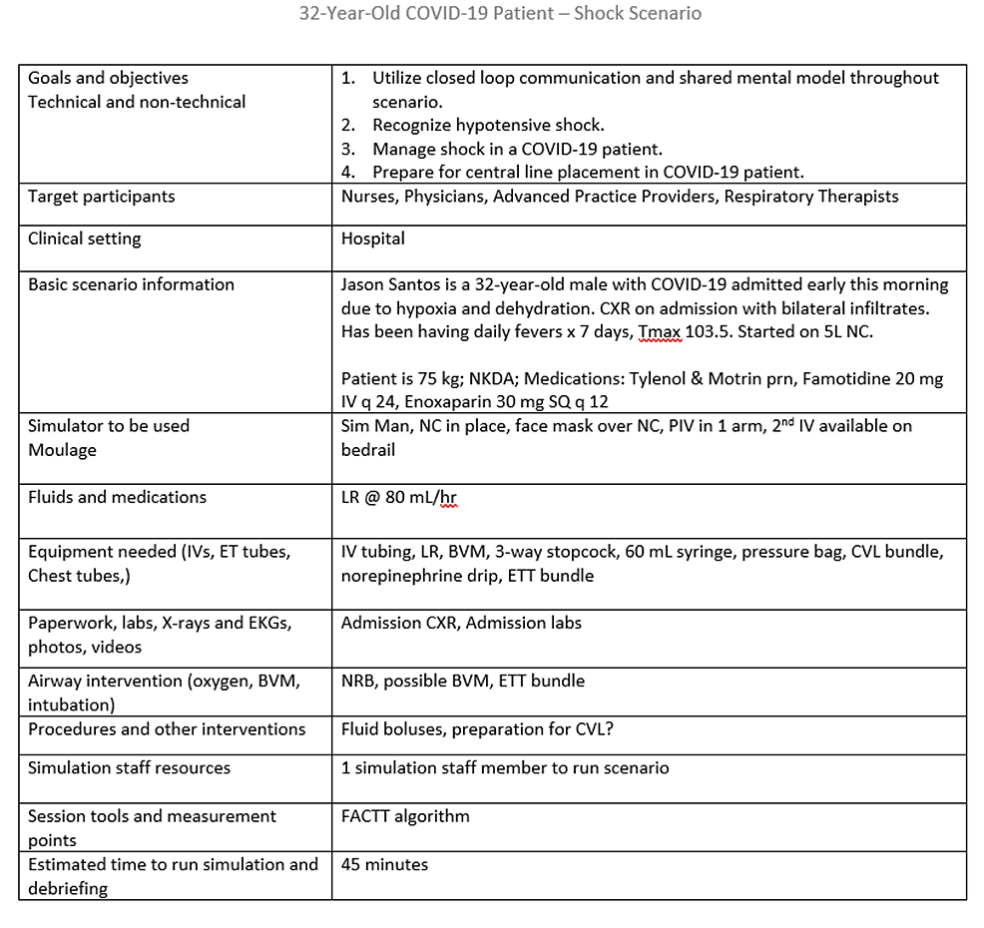
Example of scenario planning form for a Covid-19 septic shock case.

Given the setting of a “pop-up” ICU with up-trained providers and nurses, simulations were targeted around several deteriorating patients. Scenarios included septic shock, respiratory failure, and cardiac arrest; Figure 1 is an example of the planning form for the septic shock case.

**Figure 2:**
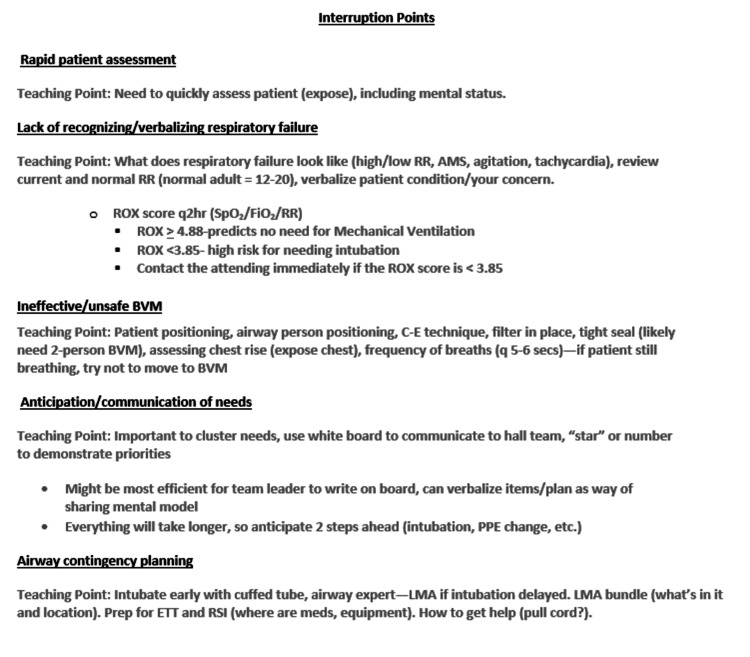
Interruption point example for a respiratory failure case. ROX = ratio of oxygen saturation, RR = respiratory rate, BVM = bag-valve-mask ventilation, PPE = personal protective equipment, LMA = laryngeal mask airway, ETT = endotracheal intubation, RSI = rapid sequence intubation

Using RCDP, we created anticipated interruption points (see Figure 2) focused on recommendations specific to adult COVID-19 patients, which varied from typical pediatric care (e.g., smaller, slower fluid boluses in shock patients). Central to every case was the use of appropriate personal protective equipment (PPE) and its concrete application to patient care.

At the time of implementation, we saw a drastic decrease in inpatient volumes, which eliminated typical scheduling obstacles for a multidisciplinary effort. We were able to bring neurosurgeons and anesthesiologists to the bedside, as well as respiratory therapists and acute care nurses. The simulations occurred in situ on the unit that would scale up if needed, which at that time was closed due to low census.

Pre-brief

Our standard pre-briefing was expanded for this training. Before starting the scenario, we introduced the reason for the training, as well as the importance of a safe learning environment. We had staff introduce themselves to one another since they often didn’t know each other and there was the possibility they would be working together in the extended ICU. Following the introductions, we reviewed the tiered care model that would exist if this was an ICU, describing the personnel and equipment resources and their locations. We discussed changes to the American Heart Association Life Support algorithms and their practical implications [3]. We introduced various communication challenges with PPE and negative pressure rooms and potential solutions. We spent time answering questions and provided an opportunity for staff to share their concerns. One of our main goals during the pre-brief was to reduce the anxiety and fear staff felt due to the unknowns of the pandemic.

Simulation and debriefing

Following the pre-briefing and mannequin introduction, each scenario started with a bedside nurse in the room with the patient. All rooms were negative pressure, so doors remained closed during the simulation. Cloth PPE was used for scenarios, and if participants left the room, they practiced doffing and re-donning PPE. The nurses escalated the situation as they deemed appropriate. Instructors interrupted the scenario at the set pause points or when other issues arose. Team members could also stop the scenario if they had questions at any moment. When the scenario was paused, the door was opened so any team members still in the hallway could participate in the discussion. Although our team created interruption points during curriculum development, the teaching points evolved with sessions. We discovered recurring themes not initially anticipated, and during the debriefing discussions, the teams helped to develop new processes. For example, at the start of the simulations, teams had a handheld whiteboard that could be held up to the window if rapid request materials were needed. During the simulations, several regularly needed items (e.g. ventilator, lactated ringers) became a large-font pre-printed checklist that could be rapidly selected and held up to the window. The RCDP model allowed us to collaborate with the teams to develop these processes and then immediately attempt and refine them. Sessions were scheduled to accommodate the total number of staff that required training. We conducted 13 sessions, each 90 minutes long and covering one of the three scenarios, over two weeks. A total of 31 providers, 42 nurses, and 11 respiratory therapists participated.

As is typical with RCDP, feedback during pauses was more directive than with traditional simulation, although there was often robust discussion during pauses as the team worked through possible solutions to their challenges. Most of these challenges centered on communication challenges with PPE and effective use of more limited personnel with COVID-19 restrictions. Post-event debriefing consisted of each participant sharing a “take away” from their experience, followed by brief highlights of key learning points by the facilitator. 

Dissemination

**Figure 3:**
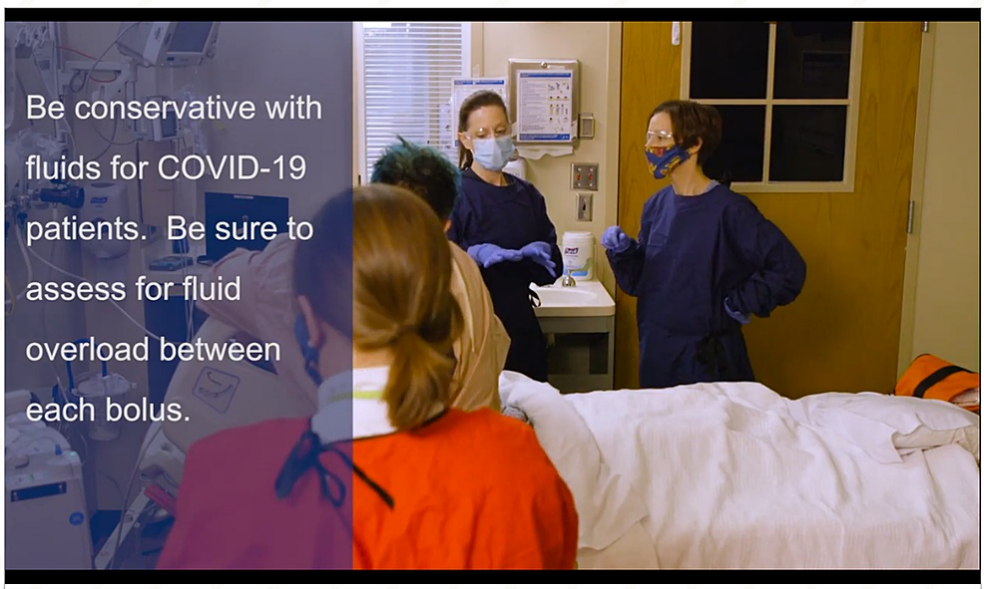
Example of teaching videos.

**Figure 4:**
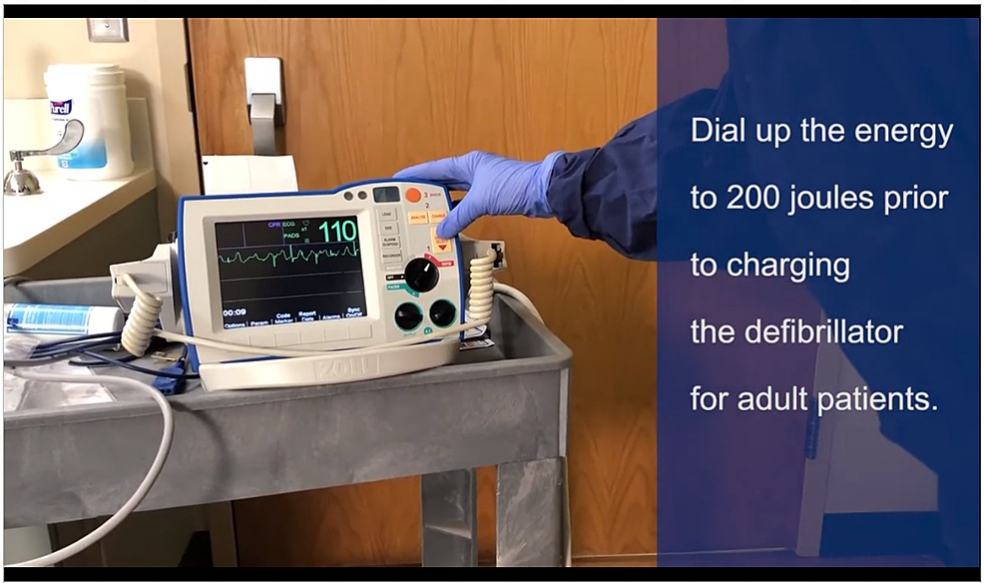
Example of teaching videos. Pertinent pearls appeared intermittently during the videos to reinforce concepts.

To date, our organization has not cared for an influx of adult COVID-19 patients. Thus, it is important to sustain and disseminate the learning that occurred. Collaborating with the audio-visual department, our simulation team video-recorded and edited all three scenarios to highlight important concepts. Figure 3 and Figure 4 are snapshots from the videos. We developed key learning points and themes from sessions which continue to be shared weekly via email with relevant personnel. These were organized into a few items per message, with links to documents and videos.

## Discussion

As with any simulation experience, it’s important to be patient, flexible, and provide a safe learning environment for the participants. The facilitators for these COVID-19 simulations identified three key themes: staff anxiety, communication and teamwork, and safety. 

The first overall theme was staff anxiety and uncertainty with the unknown. It was important to spend time pre-briefing about the logistics of the unit and the availability of equipment, personnel, and resources before beginning the simulation. Staff members were highly stressed, and we quickly identified that we could not move forward with the simulation until we decreased their anxiety, ensuring a safe learning environment [4]. We found that covering this content at the beginning allowed us to reiterate many of these key points during the pauses in the simulation. Studies performed during the COVID-19 pandemic found that simulation enhanced mental preparedness, self-efficacy, and participant’s internal locus of control, all of which could improve patient and operational outcomes [5-7].

Another common theme involved communication and teamwork. With the recommendation to minimize staff member exposures by decreasing the number of team members, we needed to identify how to provide efficient, safe patient care with smaller teams. Individuals that would typically remain in the same role throughout resuscitation needed to leave their role and do other tasks. Closed-loop communication about changing roles was important in maintaining effective teamwork. It was also challenging to communicate with PPE. We learned the use of eye contact, speaking loudly and clearly, along with incorporating name tags during the resuscitation that helped teams communicate efficiently in critical situations. Team members utilized the call-light, staff-assist button, and Voalte phones to communicate with staff outside the rooms. Many found it helpful to write messages on a dry erase board, showing it through a window to a resource person in the hall. We identified the importance of bundling care and anticipating supplies prior to entering the room, minimizing the need to open the door, and decreasing PPE use. Many aspects of patient care didn’t change with COVID-19; these new challenges helped reinforce to staff the importance of recognizing patient decline, anticipating needs, and using clear messages when communicating with new team members. 

The third theme identified was staff safety. As healthcare providers, it is not our nature to think of ourselves before the patient [8]. Ensuring staff maintained situational awareness regarding appropriate PPE and verifying all team members were safe before entering an isolation room or performing an aerosol-generating procedure was an added patient care dimension. The use of RCDP allowed us to constantly reinforce these ideas throughout a scenario.

We felt RCDP was an ideal model in which to deliver this education. One challenge we faced related to the general knowledge gaps in the medical community around optimal care for COVID-19 patients, which would have occurred regardless of the simulation modality. Perretta et al. outlined optimal settings in which to use RCDP, including the need for learners to master key behaviors which require specific scripting or choreography, specifically for low-volume, high-risk and time-sensitive situations, in the setting of limited teaching time [2]. Each of these criteria was met for training during the pandemic. 

## Conclusions

The use of RCDP allowed us to effectively train staff to function safely in a new environment. The ongoing dissemination of information is continuing to refresh and reinforce knowledge around COVID-19 protocols.
